# Structural strength of cancellous specimens from bovine femur under cyclic compression

**DOI:** 10.7717/peerj.1562

**Published:** 2016-01-25

**Authors:** Kaori Endo, Satoshi Yamada, Masahiro Todoh, Masahiko Takahata, Norimasa Iwasaki, Shigeru Tadano

**Affiliations:** 1 Department of Orthopaedic Surgery, Hokkaido University Graduate School of Medicine, Sapporo, Japan; 2 Division of Human Mechanical Systems and Design, Faculty of Engineering, Hokkaido University, Sapporo, Japan

**Keywords:** Cancellous bone, Osteoporosis, Structural indices, Collapse, Cyclic compression, Structural strength, Micro-CT

## Abstract

The incidence of osteoporotic fractures was estimated as nine million worldwide in 2000, with particular occurrence at the proximity of joints rich in cancellous bone. Although most of these fractures spontaneously heal, some fractures progressively collapse during the early post-fracture period. Prediction of bone fragility during progressive collapse following initial fracture is clinically important. However, the mechanism of collapse, especially the gradual loss of the height in the cancellous bone region, is not clearly proved. The strength of cancellous bone after yield stress is difficult to predict since structural and mechanical strength cannot be determined *a priori*. The purpose of this study was to identify whether the baseline structure and volume of cancellous bone contributed to the change in cancellous bone strength under cyclic loading. A total of fifteen cubic cancellous bone specimens were obtained from two 2-year-old bovines and divided into three groups by collection regions: femoral head, neck, and proximal metaphysis. Structural indices of each 5-mm cubic specimen were determined using micro-computed tomography. Specimens were then subjected to five cycles of uniaxial compressive loading at 0.05 mm/min with initial 20 N loading, 0.3 mm displacement, and then unloading to 0.2 mm with 0.1 mm displacement for five successive cycles. Elastic modulus and yield stress of cancellous bone decreased exponentially during five loading cycles. The decrease ratio of yield stress from baseline to fifth cycle was strongly correlated with bone volume fraction (BV/TV, *r* = 0.96, *p* < 0.01) and structural model index (SMI, *r* = − 0.81, *p* < 0.01). The decrease ratio of elastic modulus from baseline to fifth cycle was also correlated with BV/TV (*r* = 0.80, *p* < 0.01) and SMI (*r* = − 0.78, *p* < 0.01). These data indicate that structural deterioration of cancellous bone is associated with bone strength after yield stress. This study suggests that baseline cancellous bone structure estimated from adjacent non-fractured bone contributes to the cancellous bone strength during collapse.

## Introduction

Osteoporosis is a common disease which is characterized by low bone mass and deterioration of bone tissue, resulting in an increased risk of fracture, particularly at the proximity of the joint rich in cancellous bone (Riggs & Melton 3rd, 1995). Among the estimated nine million osteoporotic fractures worldwide in 2000, 1.6 million were in the hip, 1.7 million in the forearm, and 1.4 million were vertebral fractures (Johnell & Kanis, 2006). The hip and spine fractures in osteoporosis patients often include those occurring because of falls from a standing height and less due to progressive collapse. Progressive collapse is characteristically found in deformities after compress fractures with continuous loss of the height in the whole fracture bone area. For example, progressive vertebral collapse can be defined in this way: the vertebral collapse fracture is found in deformities after initial fracture, followed by continuous loss of vertebral body height. Within a femoral fracture, the occult fracture of the femur and the subchondral insufficiency fracture (SIF) of the femoral head are also manifested in the cancellous bone region with rapid progression of the collapse, such as seen in rapidly progressive arthritis of SIF (Bangil et al., 1996; Yamamoto, 2012). Severe collapse of these areas can lead to chronic pain, depression, an inability to perform daily life activities, and in extreme cases can be life-threatening (Riggs & Melton 3rd, 1995; Poole & Compston, 2006; Ioannidis et al., 2009; Adachi et al., 2010). Thus, prevention of the collapse in rich area of cancellous bone, especially proximal femur and spine fracture, is clinically critical.

Cancellous bone plays an important role as the primary load-carrying component that absorbs energy (Fyhrie & Schaffler, 1994). Characteristic stress–strain (S–S) curves indicate that the mechanical competence of cancellous bone depends mainly on the trabeculae network and material properties of the tissue. The post-yield behavior of cancellous bone has been well-documented in previous studies and linked to strain and density (Keaveny et al., 1994a; Keaveny et al., 1994b; Keaveny, Wachtel & Kopperdahl, 1999). These studies also investigated failure patterns in specimens of cancellous bone and showed similar S–S curves to those obtained. Fyhrie & Schaffler (1994) conducted on compressive tests of cancellous cubic bone and concluded that S–S curves after linear region were considered to have three phases which correspond with: (1) the initial trabeculae yield; (2) the secondary trabeculae yield and buckle; and (3) the plateau region. Mechanical properties in three phases decreased and finally plateaued, while the strain increased and the height loss progressively collapsed in areas rich with cancellous bone without specific trauma.

According to macroscopic experiments using compression tests, the stiffness of cancellous bone is correlated with its apparent density (Keller, 1994; Morgan & Keaveny, 2001; Morgan, Bayraktar & Keaveny, 2003; Follet et al., 2004). In addition, mechanical properties that contribute to bone strength are determined by bone geometry (size and shape of trabecular bone) and microarchitecture using noninvasive three-dimensional micro-computed tomography (micro-CT) evaluation (Kopperdahl & Keaveny, 1998; Ulrich et al., 1999; Ahlborg et al., 2003; Todoh et al., 2004). However, some studies suggest that detection of bone geometries and microarchitecture, as well as structural indices, is associated with fracture in the distal radius and tibia (Genant et al., 1996; Mackey et al., 2007; Link & Lang, 2014). Therefore, the geometry and microarchitecture of cancellous bone contributes to the bone structural strength and would likely enable estimation of osteoporotic fracture risk.

However, the means by which the mechanical properties of cancellous bone were decreasing after the elastic region, and which structural factors affected the decreased mechanical properties, has not been well documented. This is because the non-linear response of cancellous bone post-yielding is more difficult to interpret than the linear response of the cortical diaphysis. In addition, the thin cortical shell covers 45% of the mid-transverse section to as low as 15% at the endplates (Eswaran et al., 2006). To advance our knowledge of the mechanism of progressive collapse, it is necessary to understand functional adaptation of cancellous bone to post-yield stress tendency. Moreover, identifying the relationship between the structural indices of cancellous bone and decreased mechanical properties post-yield may contribute to understanding the gradual decrease of the height in progressive collapse. Hence, the aim of this study was to investigate how cancellous bovine bone specimens taken from different areas of the femur change in elastic modulus and yield stress during cyclic compressions, and to examine the relationships between structural indices and decreased strength after the elastic region.

## Methods

### Specimen preparation

**Figure 1:**
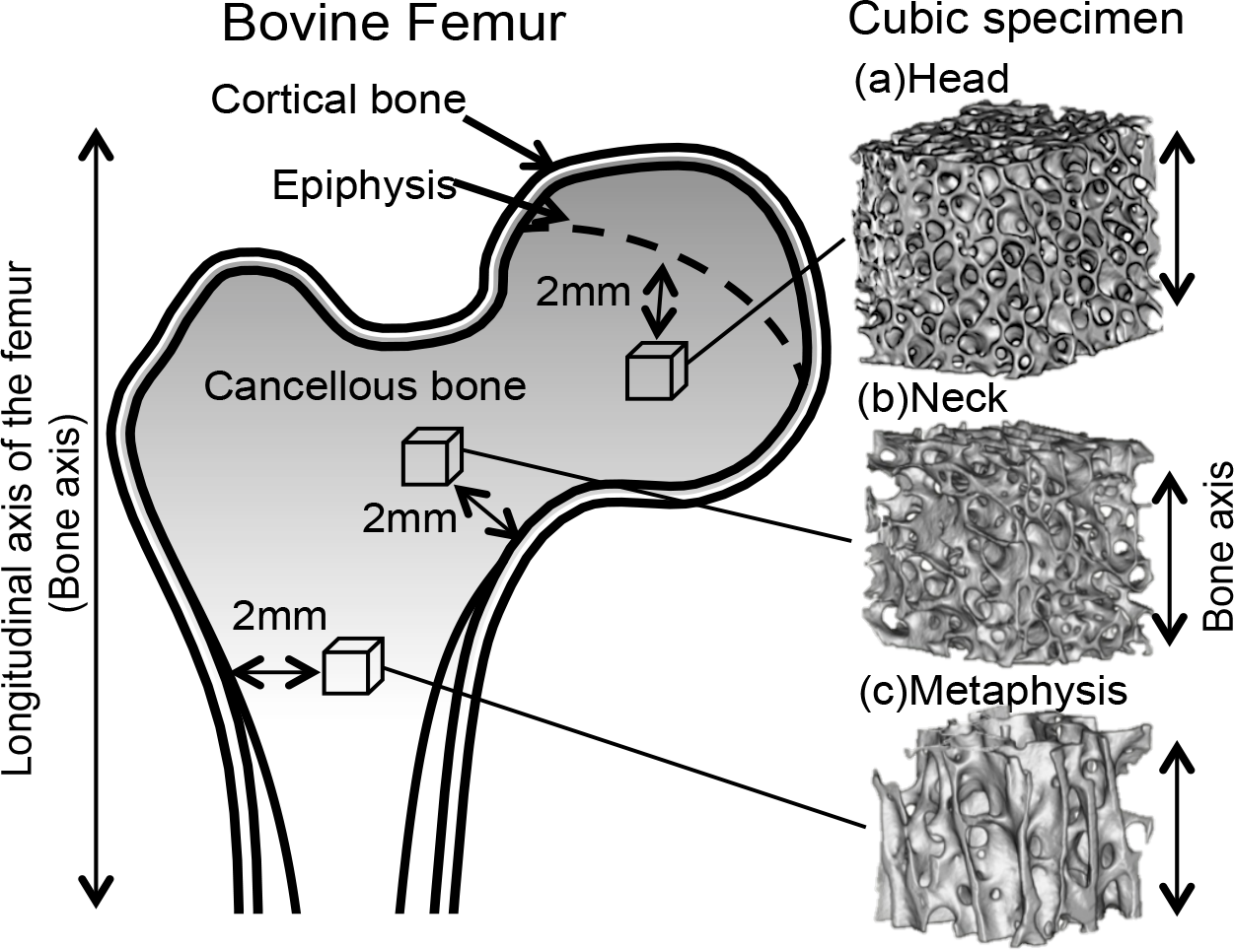
Cubic specimens were obtained along the bovine femur bone axes from the (A) head, (B) neck, and (C) metaphysis.

A total 15 specimens were extracted from two bovines (A and B); 8 specimens were obtained from the right femur of bovine A and 7 specimens were obtained from the left femur of bovine B as shown in Table 1. Specimens were grouped (*n* = 5∕groups) in accordance with their extraction regions: the proximal metaphysis, neck, and head (Fig. 1). The femurs were first sliced along the coronal plane and then into 5-mm cubic shapes using a diamond wheel saw (model 650; South Bay Technology, San Clemente, CA, USA). All specimens containing cancellous bone were retrieved where one axis of the specimen corresponded to the longitudinal axis and 2 mm away from the cortical bone and epiphysis. Bone marrow was removed from the specimens using a pressure water jet, brushes, and automatic ultrasound device (US-1; Samsung, Tokyo, Japan). Specimens were stored in a container at −35 °C until experimentation. Preliminary experiments demonstrated that the bone volume fraction (BV/TV) increased in direct proportion with the proximity of the specimen from the femur metaphysis to the head. In accordance with this finding, specimens were grouped into three parts according to BV/TV, differing by approximately 10% between each part, as shown in Table 1.

**Table 1:** Mechanical properties and structural indices are shown. These data are shown as mean and standard deviation.

Specimen			Mechanical properties		Structural indices									tBMD (g/cm^3^)
Area	No	Bovine	Yield stress *σ*_*Y*1_(MPa)	Elastic modulus *E*_1_(MPa)	BV/TV(%)	Connectivity	SMI	DA	Fractal dimension	Tb.Th. (mm)		Tb.Sp. (mm)		
										Mean	S.D.	Mean	S.D.	
Meta-physis	1	A	14.2	435.5	20.6	182	1.66	0.79	2.45	0.24	0.07	1.19	0.45	732.9
	2		22.3	504.0	24.4	111	1.19	0.80	2.49	0.28	0.09	0.99	0.35	774.3
	3	B	15.6	460.3	14.2	238	2.78	0.73	2.47	0.17	0.04	0.83	0.27	692.2
	4		18.2	314.0	22.6	175	1.47	0.77	2.44	0.27	0.08	1.00	0.38	768.3
	5		13.6	426.9	24.3	162	1.38	0.71	2.57	0.23	0.06	0.85	0.32	735.3
	Mean		16.8	428.1	21.2	173	1.70	0.76	2.48	0.24	0.07	0.97	0.35	740.6
	S.D.		3.6	70.5	4.2	45	0.63	0.04	0.05	0.04	0.02	0.15	0.07	32.9
Neck	6	A	19.7	763.0	29.1	659	2.02	0.62	2.47	0.20	0.06	0.81	0.29	675.3
	7		16.5	518.6	40.6	272	1.46	0.62	2.72	0.24	0.07	0.65	0.20	701.3
	8		16.3	462.6	40.7	804	1.21	0.52	2.69	0.25	0.06	0.69	0.25	683.3
	9	B	9.4	237.8	35.0	783	1.79	0.66	2.66	0.22	0.06	0.73	0.26	688.4
	10		18.1	620.7	40.0	1,074	0.86	0.75	2.59	0.26	0.09	1.12	0.76	692.4
	Mean		16.0	520.5	37.1	718	1.47	0.63	2.63	0.23	0.07	0.80	0.35	688.1
	S.D.		3.9	195.0	5.1	292	0.46	0.08	0.10	0.02	0.01	0.19	0.23	9,8
Head	11	A	30.1	871.8	47.6	10,736	−0.64	0.85	2.80	0.18	0.08	0.29	0.18	726.8
	12		26.9	779.0	48.6	8,039	−2.31	0.85	2.82	0.20	0.08	0.31	0.19	722.8
	13		30.4	1,022.7	48.9	874	−1.64	0.70	2.81	0.23	0.05	0.45	0.18	724.3
	14	B	31.1	970.2	47.0	1,459	−0.88	0.74	2.85	0.23	0.06	0.41	0.16	716.0
	15		31.4	990.6	51.9	9,656	−1.79	0.64	2.88	0.19	0.08	0.32	0.17	709.4
	Mean		30.0	926.9	48.8	6,152	−1.45	0.75	2.83	0.21	0.07	0.35	0.18	719.9
	S.D.		1.8	100.0	1.9	4,657	0.68	0.09	0.03	0.02	0.01	0.07	0.01	7.1
All area	Mean		20.9	625.2	35.7	2,348	0.57	0.72	2.65	0.22	0.07	0.71	0.29	716.2
	S.D.		7.2	258.9	12.2	3,150	1.61	0.08	0.15	0.03	0.01	0.27	0.15	28.3

### Microstructure analysis using micro-CT

The micro-CT technique and image processing approach were based on current guidelines and a previous study (Bouxsein et al., 2010). All specimens were scanned using a micro focus CT instrument (InspeXio SMX-90CT; Shimadzu, Kyoto, Japan) at a 22 µm horizontal grid spacing and slice interval of 90 kV and tube current of 110 mA. Tissue bone mineral density (tBMD) was calibrated from the gray scale linear value using an imaging reference micro-CT phantom. A review of the literature indicated that filtration does not necessarily require software-based beam hardening corrections for imaging of cancellous bone (Waarsing, Day & Weinans, 2004; Meganck et al., 2009). In preliminary assessments, a cover of only 0.1 mm Cu as an X-ray filter could reduce the induced beam hardening enough to calculate tBMD and structural indices. To assess the volumetric density and microarchitecture of cancellous bone, Bone J software can be used to calculate a number of structural indices (Doube et al., 2010). The structural indices determined in this study included BV/TV, degree of anisotropy (DA), trabecular thickness (Tb.Th.), trabecular space (Tb.Sp.), structure model index (SMI), and number of connections (connectivity).

### Cyclic compression test

**Figure 2:**
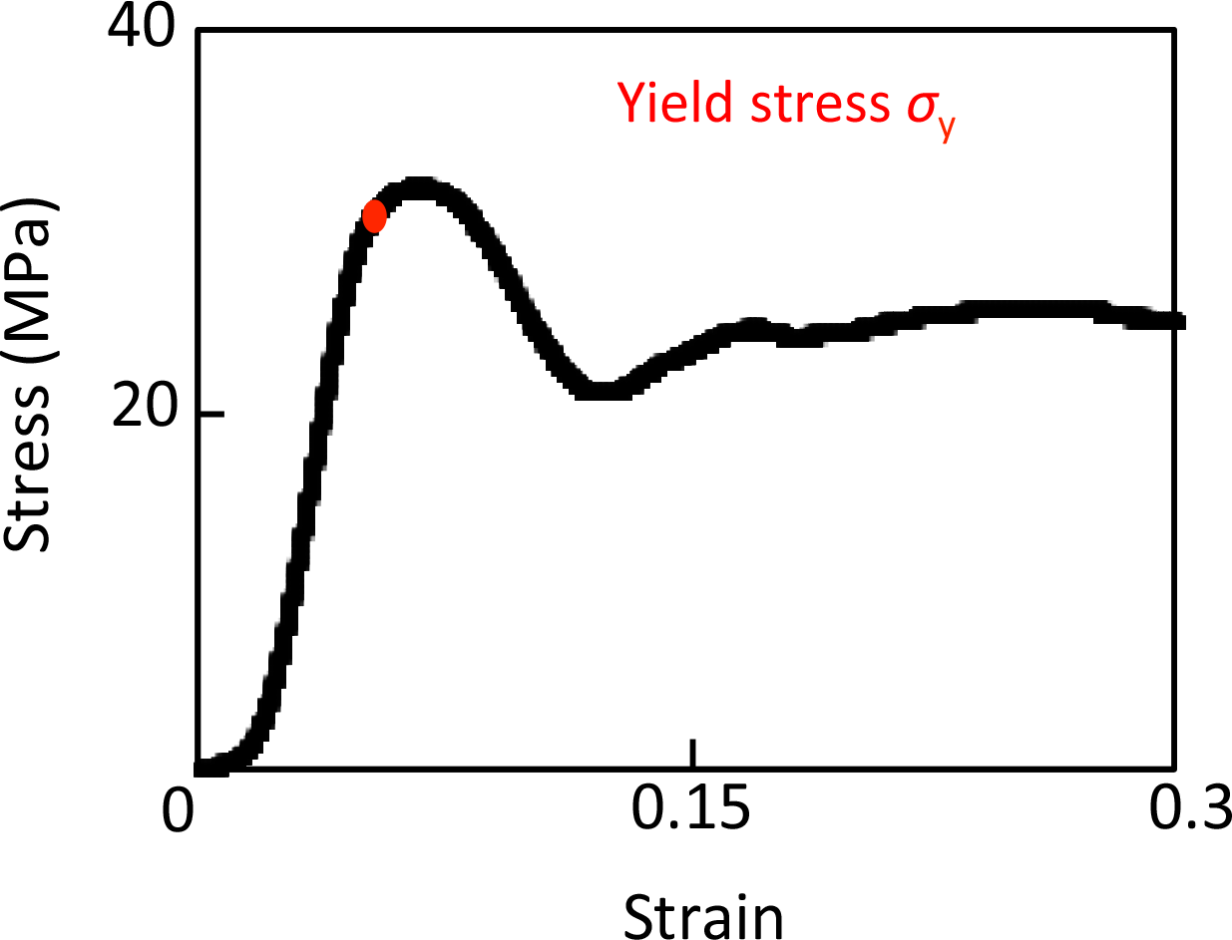
Stress–strain curve of successful uniaxial compression.

Uniaxial compression tests were conducted on the specimens in the longitudinal direction to measure the apparent elastic modulus and yield stress using a mechanical testing machine (model 3365; Instron, Grove City, PA, USA) at room temperature. Loading parameters were established during preliminary experiments using bone samples. Figure 2 shows that the S–S curves obtained in this experiment also had the three phases described previously in the introduction (Fyhrie & Schaffler, 1994). A strain of 0.06 (0.3 mm displacement) was chosen to ensure failure of the three specimens in the head groups, as this exceeds the failure strain by an average 1.5 times.

**Figure 3:**
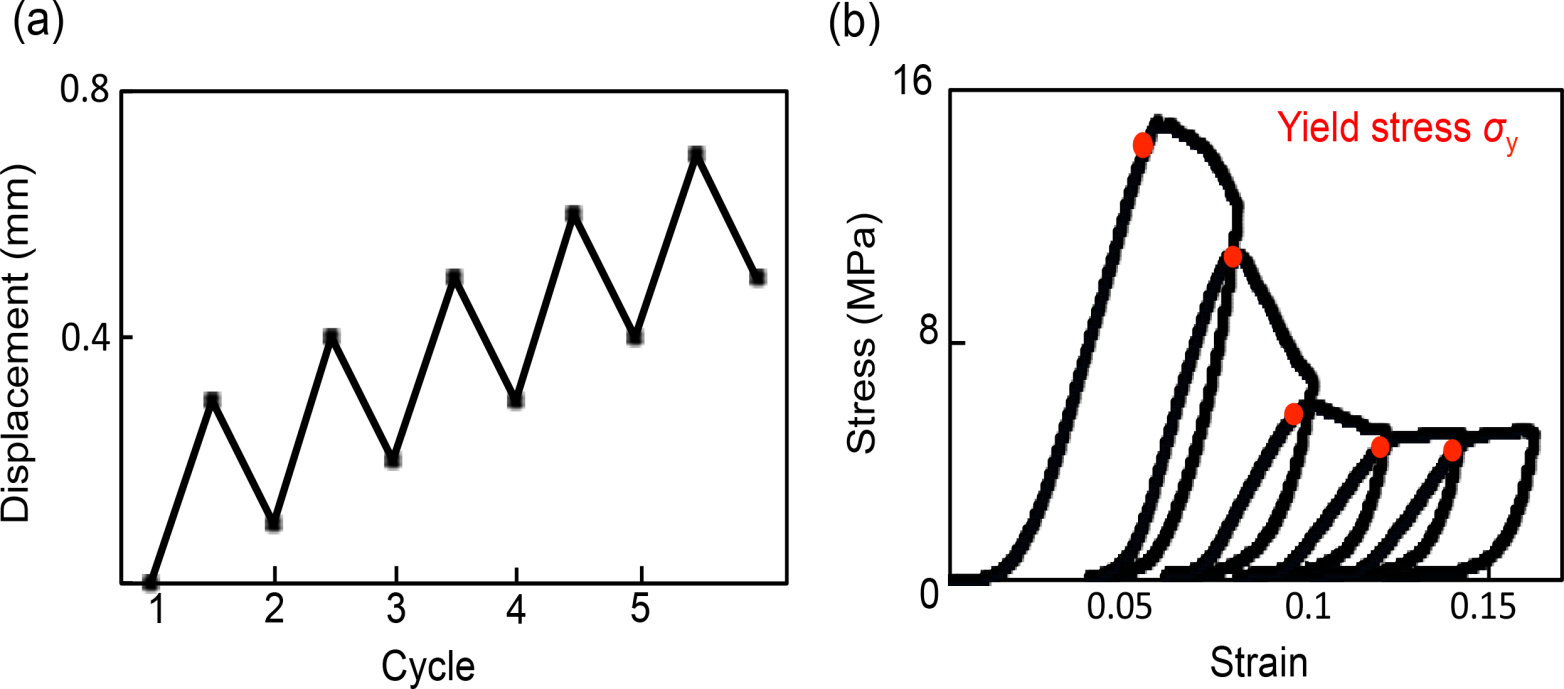
Method and stress–strain curves of cyclic compression. (A) Total displacement of each cycle increased with 0.1-mm displacement. (B) Stress–strain curves under cyclic compression; yield stress was set to the maximum point, and elastic modulus were calculated from 50% to 70% of the maximum load in each cycle.

Finally, the optimal experimental conditions for cycles of loading were identified as follows: 0.3 mm displacement then unloading to 0.2 mm with 0.1 mm displacement for five successive cycles (Fig. 3A). The five compression cycles were set to auto control regulation with preloading of 20 N and a displacement rate of 0.05 mm/min. The apparent elastic modulus was calculated from 50% to 70% of maximum loading in each cycle. Yield stress was defined as the inflection point that existed between the linear region to the plateau or the stress decreasing region (Fig. 3B).

### Statistical analysis

Pearson’s correlation coefficient between the mechanical properties and structural indices were calculated and considered to be significant at *p* < 0.01. JMP Pro software (Version 11.0.0; SAS Institute Inc., Cary, NC, USA) was used to perform statistical analyses.

**Figure 4:**
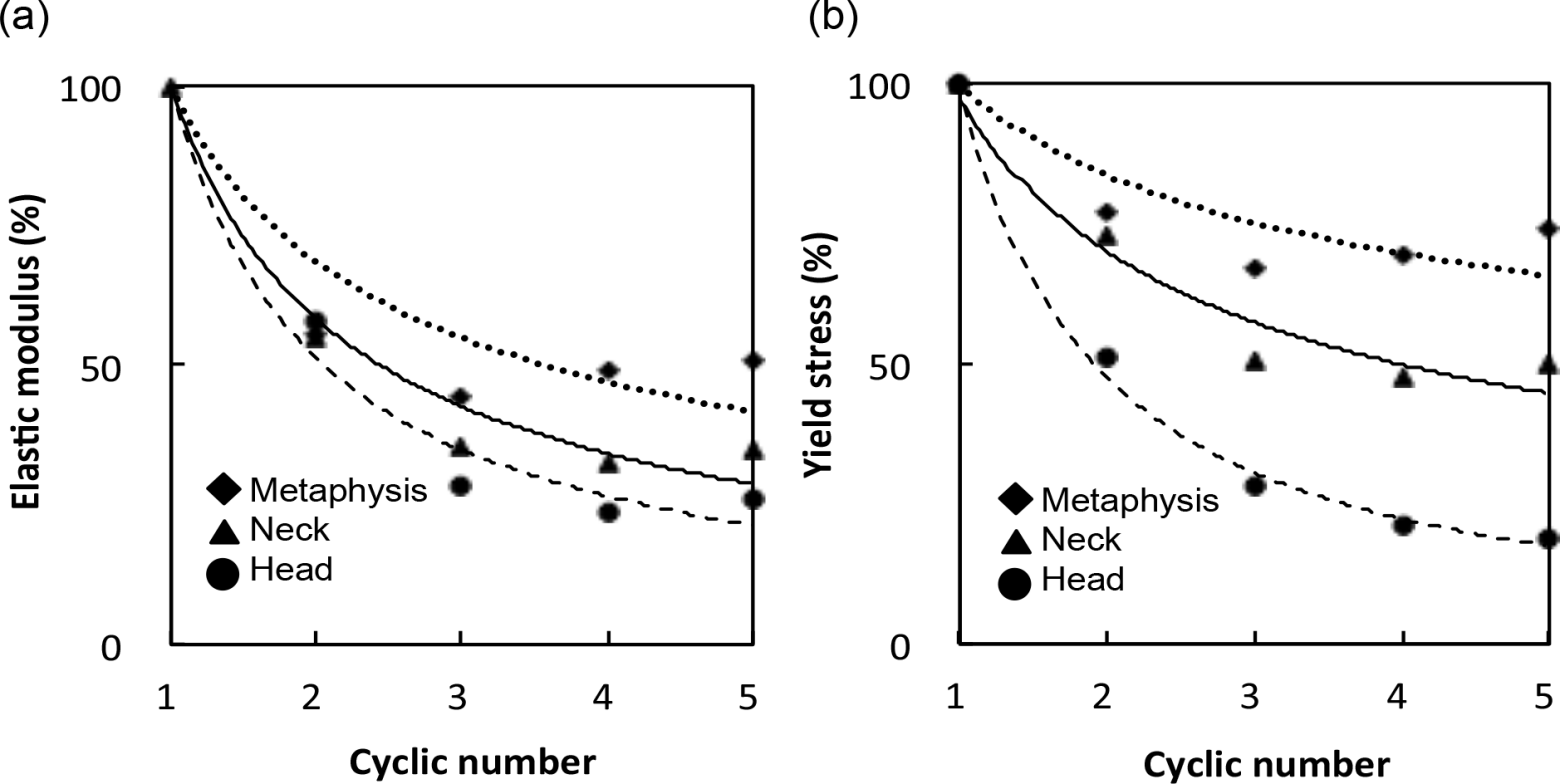
(A) Elastic modulus and (B) yield stress of each cycle are expressed relative to those of the first cycle.

## Results

Mechanical properties, structural indices and tBMD of the specimens are shown in Table 1. BV/TV correlated negatively with Tb.Sp.Mean and positively with connectivity. SMI identified plate, rod and concave shapes in the trabeculae of the metaphysis, neck and head groups, respectively. There were no significant differences with respect to the mean DA and Tb.Th between the groups. However, elastic modulus was significantly different between the groups after the first cycle, with values of 428.1 ± 70.5 MPa in the metaphysis group, 520.5 ± 195.0 MPa in the neck group, and 926.9 ± 100.0 MPa in the head group. Yield stress at the first cycle was 16.8 ± 3.6 MPa in the metaphysis group, 16.0 ± 3.9 MPa in the neck group, and 30.0 ± 1.8 MPa in the head group. Bone strength was considerably reduced after the first yield stress, and then decreased more moderately with subsequent cycles (Fig. 4).

**Figure 5:**
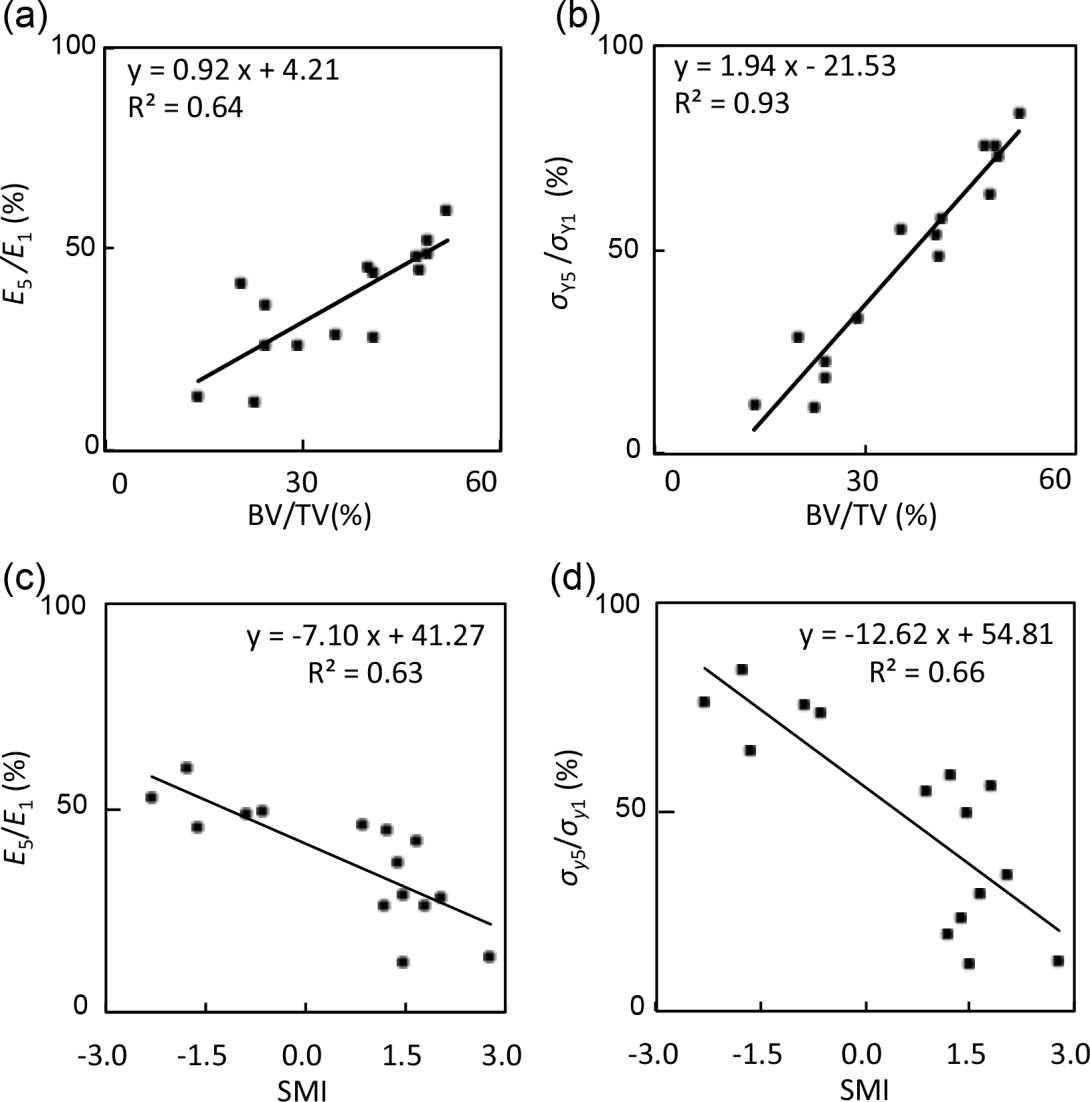
Correlations between mechanical properties and structural indices. These graphs showed (A) *E*_5_∕*E*_1_ and BV/TV (*r* = 0.80), (B) *σ*_*Y*5_∕*σ*_*Y*1_ and BV/TV (*r* = 0.96), (C) *E*_5_∕*E*_1_ and SMI (*r* = − 0.78), and (D) *σ*_*Y*5_∕*σ*_*Y*1_ and SMI (*r* = − 0.81).

The elastic modulus ratio between the fifth and first cycle (*E*_5_∕*E*_1_) was 26.0 ± 13.4% in the metaphysis group, 34.7 ± 9.7% in the neck group, and 50.9 ± 5.5% in the head group. Moreover, the yield stress ratio between the fifth and first cycle (*σ*_*Y*_5__∕*σ*_*Y*_1__) was 18.9 ± 7.3% in the metaphysis group, 49.7 ± 9.7% in the neck group, and 74.3 ± 7.1% in the head group. Table 2 showed that BV/TV, connectivity, SMI, and Tb.Sp.Mean were significantly correlated with mechanical properties (*E*_1_ and *σ*_*Y*_1__), and with the decrease ratio (*E*_5_∕*E*_1_ and *σ*_*Y*_5__∕*σ*_*Y*_1__), though the majority of parameters were poorly correlated. The strongest correlations were found between SMI and *σ*_*Y*1_ (*r* = − 0.86) and *E*_1_ (*r* = − 0.67). Figure 5 showed that BV/TV was strongly correlated with *E*_5_∕*E*_1_ (*r* = 0.80) and *σ*_*Y*_5__∕*σ*_*Y*_1__ (*r* = 0.96). SMI was negatively correlated with *E*_5_∕*E*_1_ (*r* = − 0.78) and *σ*_*Y*_5__∕*σ*_*Y*_1__ (*r* = − 0.81). *σ*_*Y*_5__∕*σ*_*Y*_1__ was also correlated with connectivity (*r* = 0.66) and Tb.Sp.Mean (*r* = − 0.79).

**Table 2:** Correlation coefficients between structural indices and mechanical properties.

	DA	BV/TV	Connectivity	SMI	Tb.Th mean	Tb.Sp mean
*E*_1_	−0.08	**0.63** ^*^	0.45	−0.67^*^	0.29	**−0.67** ^*^
*σ*_*Y*1_	0.06	**0.69** ^*^	0.34	**−0.86** ^*^	0.37	**−0.71** ^*^
*E*_5_∕*E*_1_	−0.47	**0.80** ^*^	0.38	**−0.78** ^*^	0.37	−0.55
*σ*_*Y*5_∕*σ*_*Y*1_	−0.43	**0.96** ^*^	**0.66** ^*^	−0.81^*^	0.26	**−0.79** ^*^

**Notes.**

* *p* < 0.01.

## Discussion

The present study elucidated two important post yielding mechanical phenomena of cancellous bone in order to understand the progressive collapse. Firstly, both elastic modulus and yield stress initially decreased rapidly and then decreased more gradually in line with the exponential approximation. In addition, both of the decrease ratios of elastic modulus and yield stress were inversely proportional to BV/TV. Following the exposure of cancellous bone to compressive loads high enough to initiate buckling of the trabeculae, mechanical strength decreases and progressive collapse occurs much easier in groups with low BV/TV. Thus, breakage of the trabeculae following load stress may impair the strength of the cancellous bone. From this viewpoint, the collapse in osteoporosis patients might progress rapidly as a consequence of the significant decrease of cancellous bone strength. Secondly, the mechanical properties at the first cycle and the decrease ratio correlated strongly with BV/TV and SMI, and weakly with connectivity and Tb.Sp.Mean. The novel relationships between the decrease ratio of mechanical properties and structural indices allowed estimations of the decrease in mechanical strength. Hence, these results indicated that baseline cancellous bone structure could help to understand cancellous bone strength during collapse. It has recently been shown that clinical CT can determine structural indices with a resolution of 0.1 mm (Burghardt et al., 2007). This study suggests that baseline bone structure estimated from the adjacent non-fractured bone side can contribute to the cancellous bone strength during clinical collapse. This bone fragility of cancellous bone during progressive collapse is only one part of the whole bone strength; however, medical institutions could more correctly evaluate the permissible pressure of the fracture area and more confidentially allow each osteoporosis patient to undertake the appropriate levels of rehabilitation without progressive collapse.

This first experiment in terms of using cyclic compression testing helps to understand the post-yield tendency of progressive collapse in areas rich with cancellous bone. Additionally, our study used successive compression testing which increased strain in every cycle. Keaveny et al. (1994b) aimed at determining how the modulus and strength decreased after being subjected to yield stress with various initial loading strains. Figure 4 shows that the approximate amount of decrease ratio in our study is similar in elastic modulus but different in yield stress compared to their study. The reason for this may be because of differences in the specimens preparations, experimental settings or in cycle numbers.

The bovine femurs were suitable for the purpose of this study because they had vast differences in the microarchitecture, especially with respect to BV/TV. Our study highlighted that the bovine femurs included the distal cancellous bone comprising the metaphysis with a lower BV/TV of the whole femur, and the proximal end of femur comprising the femoral head with a higher BV/TV. Compared with previous studies that have also identified BV/TV as a strong predictor of bone strength, our study examines a range of BV/TV four times wider than those in other studies using identical conditions (Goulet et al., 1994; Dux et al., 2010; Slyfield et al., 2012; Hernandez et al., 2014). Other studies have also included a broader range of BV/TV, but under different preparations; for example, after nephrectomy, ovariectomy or loading (Cory et al., 2010; Morgan, Bostrom & Van der Meulen, 2015). Destructive mechanical tests would be more likely to result in complicated S–S curves. Limiting the focus of structural indices would simplify interpretations superior in accuracy to prevent errors occurring from other factors. Therefore, the novelty of our study compared to other studies is broad range of BV/TV with the same preparations.

For further improvements towards more accurate estimation of cancellous bone strength in our *in vitro* model, other additional points were considered. First, the mechanical properties of single trabeculae consisting of cancellous bone had to be considered. Yamada, Tadano & Fukuda (2014) previously reported on single trabecular strength in bovine femurs and the relationship between mechanical properties and nanostructure. To more accurately predict bone strength, the structural strength of the whole cancellous bone was applied in our work, with fine distinctions in material strength also applied using their technique. Second, many studies (Tang & Vashishth, 2010; Lambers et al., 2013; Hernandez et al., 2014; Lambers et al., 2014) reported that the damaged bone fraction affected elastic modulus and yield stress.Use of these microscopic techniques to detect the damaged bone fraction might anticipate the three phases in post-yielding. More specifically the second and third phases, being the secondary trabecular yield and buckle and the plateau region, respectively. In fact, our experiments attempted to understand the process of trabecular buckling using micro-CT imaging, but obvious trabecular buckling was not observed from the first yield to the start of plateau region. In future experiments, measuring the damaged bone fractions might help to interpret the mechanism of decreased bone strength after the first trabecular yielding in our experiments.

There were two limitations; the first limitation was that this study selected the direction of compression only in the longitudinal direction. Previous studies have also used cubic specimens from various parts and animals but have compared the elastic modulus compressed in three directions (Vahey, Lewis & Vanderby Jr, 1987; Goulet et al., 1994; Frich et al., 1997; Morgan, Bostrom & Van der Meulen, 2015). These studies showed that the elastic modulus in the gravity direction was between 2 and 4 times greater than in the other directions. Conversely, the strongest elastic modulus in our two bovine specimens was not necessarily along the gravity direction as the specimens were relatively isotropic. The uniaxial compression tests of the cubic specimens were conducted in each of three directions and the maximum elastic modulus divided by minimum elastic modulus was 1.57 ± 0.47. This study also showed that the degree of anisotropy was not correlated with the mechanical properties in cases where the specimens were limited to bovine femurs. Therefore, this study ignored the effect of compression direction. For subsequent investigations, we will consider how the mechanical properties of the cancellous bone are influenced by the main trabecular direction of the human anatomical bone microstructure, using the mean intercept length method (Odgaard et al., 1997; Perilli et al., 2008).

The second limitation was that the low number of specimens (*n* = 15) obtained from only two young bovines with epiphysis. However, the latter limitation was also the scheme of our study to minimize the complicated errors in establishing the relationship between structural indices and the decrease ratio of mechanical properties at first. The reason was that healthy and young bovines tend to have little risk of secondary osteoporosis. In future studies we will need to consider other complex factors such as BMD, collagen quality and bone turnover. The experimental conditions must be achieved by a carefully designed *in vivo* study and partly referring to our data of bovine specimens in this study. Other studies have shown that the elastic modulus and yield stress in some patients are not compromised by reduced BMD; for example, in severe osteoporosis, metastasis (including after irradiation), necrosis, and secondary osteoporosis (Lavernia, Sierra & Grieco, 1999; Fazzalari et al., 1998; Wu et al., 2008; Dux et al., 2010; Keaveny et al., 2014). Hence, structural indices have a great potential to become disease-specific predictors. In the future, structural indices might become the first predictors for the risk of progressive collapse in rich area of cancellous bone after an initial fracture to enable better treatments, external fixation, and more appropriate levels of activity and rehabilitation.

## Conclusions

Elastic modulus and yield stress were significantly decreased at the first trabeculae yield, and then decreased more gradually as the number of compression cycles was increased. Subsequent decrease ratio in elastic modulus and yield stress was significantly correlated with bone volume fractions and connectivity of cancellous bone. These results contribute to one of the predictions of cancellous bone strength during progressive collapse, from baseline cancellous bone structure estimated from the adjacent non-fractured side bone.

## Additional Information and Declarations

### Data Availability

The following information was supplied regarding data availability: The raw data can be found in Table 1.
